# Antibiotic prophylaxis with teicoplanin on alternate days reduces rate of viridans sepsis and febrile neutropenia in pediatric patients with acute myeloid leukemia

**DOI:** 10.1007/s00277-016-2833-5

**Published:** 2016-10-04

**Authors:** Heidrun Boztug, Nora Mühlegger, Ulrike Pötschger, Andishe Attarbaschi, Christina Peters, Georg Mann, Michael Dworzak

**Affiliations:** St. Anna Kinderspital and Children’s Cancer Research Institute, Department of Pediatrics, Medical University of Vienna, Kinderspitalgasse 6, 1090 Vienna, Austria

**Keywords:** Acute myeloid leukemia, Children, Antibiotic prophylaxis, Viridans streptococci, Febrile neutropenia

## Abstract

Intensive chemotherapy directed against acute myeloid leukemia of childhood is followed by profound neutropenia and high risk for bacterial and fungal infections, including viridans group streptococci as a common cause for gram-positive septicemia. Few retrospective studies have shown the efficacy of various antibiotic prophylactic regimens in children. We retrospectively studied 50 pediatric patients treated on the AML-BFM 2004 protocol between 2005 and 2015 at St. Anna Children’s Hospital and assessed the effect of antibiotic prophylaxis on the frequency of febrile neutropenia and bacterial sepsis. Fifty pediatric patients underwent 199 evaluable chemotherapy cycles. Viridans sepsis occurred after none of 98 cycles with prophylactic administration of teicoplanin/vancomycin in comparison to 12 cases of viridans sepsis among 79 cycles without systemic antibacterial prophylaxis (0 vs. 15 %, *p* < 0.0001). In addition, there were significantly fewer episodes of febrile neutropenia in the teicoplanin/vancomycin group (44 % vs. no prophylaxis 82 %, *p* < 0.0001). Severity of infection seemed to be worse when no antibiotic prophylaxis had been administered with a higher rate of intensive care unit treatment (0/98, 0 %, vs. 4/79, 5 %, *p* = 0.038). So far, no increase of vancomycin-resistant enterococcus isolates in surveillance cultures was noticed. Antibiotic prophylaxis with teicoplanin (or vancomycin) appears safe and feasible and resulted in eradication of viridans sepsis and decreased incidence of febrile neutropenia in pediatric AML patients. The possibility to administer teicoplanin on alternate days on an outpatient basis or at home could contribute to patient’s quality of life and decrease health care costs.

**Electronic supplementary material:**

The online version of this article (doi:10.1007/s00277-016-2833-5) contains supplementary material, which is available to authorized users.

## Introduction

Acute myeloid leukemia (AML) of childhood and adolescence accounts for 20 % of pediatric leukemias. Cure rates are lower in comparison to those in acute lymphoblastic leukemia [1]. Yet, intensive chemotherapy based mainly on anthracyclines and purine analogues has improved outcome over the last decades [1–3]. Profound and prolonged neutropenia following intensive chemotherapy cycles, however, is associated with a high risk for bacterial and fungal infections contributing to considerable therapy-associated morbidity and mortality [4, 5]. Among the different pediatric AML study groups, the main cause of treatment-related death following induction is infection—between 5 and 15 % of the patients have been reported to die from infectious complications [6–8]. Viridans group streptococci (VGS) are a common cause for sepsis and pneumonia in neutropenic patients. According to data from study AML-BFM 93 and 2004, VGS was isolated in more than 20 and 30 % of cases with neutropenic bacteremia, respectively. While incidence of VGS sepsis was significantly higher after chemotherapy cycles containing high-dose cytarabine in protocol AML-BFM 93, this difference was not obvious in the 2004 study [4, 9]. Infection with VGS bears substantial risk for viridans streptococcal shock syndrome with a reported mortality rate of up to 100 % [10]. High morbidity and mortality associated with neutropenic infections have caused long-standing discussions about antibiotic prophylactic regimens for pediatric AML patients [11]. At St. Jude Children’s Research Hospital, a prophylactic regimen of intravenous (iv) cefepime or iv vancomycin plus either oral cephalosporine, oral cipropfloxacine or iv cefepime has been applied to neutropenic pediatric AML patients [12]. A reduction of (VGS) septicemia and hospitalization days could be demonstrated with this approach; however, incidence of febrile neutropenia was similar, as recently published in an update to the original study [12, 13]. As vancomycin has to be administered at least every 12 h iv to obtain adequate blood levels, this prophylactic regimen either requires hospitalization or at-home administration by trained caregivers.

Teicoplanin is a glycopeptide antibiotic with long elimination half-life that is usually administered once daily. However, an alternate-day dosing schedule with administration three times a week (for example, Monday-Wednesday-Friday) on an outpatient basis has been studied in patients with chronic osteomyelitis or endocarditis and resulted in comparable and adequate serum concentrations [14–16].

We report now our results on the use of prophylactic outpatient administration of teicoplanin (or vancomycin in case of admission to hospital) in neutropenic pediatric AML patients in order to reduce febrile neutropenia, bacterial sepsis, and VGS sepsis, in particular.

## Materials and methods

### Patients

Between May 2005 and July 2015, 50 consecutive pediatric patients with AML (male *n* = 25, age 0.2–17.9, median 7.6 years) were enrolled into trial AML-BFM 2004 at St. Anna Children’s Hospital in Vienna. The initial diagnosis of AML and assignment to subtypes were done according to the FAB and WHO classification (FAB M0 *n* = 3, M1 *n* = 2, M2 *n* = 16, M3 *n* = 7, M4 *n* = 6, M5 *n* = 8, M7 *n* = 6, NA *n* = 2—one patient with myelosarcoma, one with secondary AML) [17, 18]. After confirmation of diagnosis, parental or patient informed consent for data registration and follow-up was obtained in all patients according to local laws and regulations. All investigations had been approved by the Institutional Ethics Committees. We performed a retrospective chart review of these 50 patients and collected data on use of antibiotics as well as incidence and severity of infection. Data were not collected after relapse or stem cell transplant. Courses during which a patient received antibiotics at the start of chemotherapy due to documented infection (or suspected infection in case of nonneutropenic fever along with chemotherapy) were excluded from our analysis.

### AML treatment

The aim of AML-BFM 2004 protocol (Eudract-Nr. 2006-004710-41, NCT00111345) was to evaluate in two randomizations if the prognosis of childhood AML could be further improved by therapy intensification (see supplemental Table 1 for chemotherapy details). Liposomal daunorubicin was implemented in first induction in a theoretically higher dosage than idarubicin (first randomization, course ADxE), and 2-CDA was evaluated as intensification in consolidation therapy for high-risk (HR) patients (second randomization, course AI/2-CDA) [19]. In May 2010, randomizations were closed and all patients received induction with ADxE and consolidation with AI/2-CDA in the HR group [20]. Patients with Down syndrome were treated according to standard risk (SR) group; however, induction with AIE was continued and etoposide in intensification (HAE) was omitted. Cytarabine dose varied between the different courses (see supplemental Table 1): while a low dose of cytarabine was administered during induction (ADxE, AIE: total dose of 1.400 mg/m^2^, first 48-h continuous administration, then every 12 h), patients received high-dose cytarabine during courses HAM, HAE, and HA (total dose 18.000 mg/m^2^). In course haM, an intermediate dose of 6000 mg/m^2^ cytarabine (1000 mg every 12 h over 3 days) was administered. The total dose of cytarabine in courses AI and AI/2-CDA was 2000 mg/m^2^; however, this dose was administered as a continuous infusion of 500 mg/m^2^/day over 4 days. Previous studies have shown that cytarabine as continuous infusion is associated with higher antileukemic efficacy but also increased grade 3/4 toxicity rate in comparison to daily short-term infusion [21]. Hence, we combined cycles haM and AI/AI/2-CDA to the intermediate dose group for analysis.

### Supportive care

Different prophylactic antibiotic regimens were used within the study period at St. Anna Children’s Hospital to prevent treatment-associated bacterial sepsis in general and VGS sepsis in particular. While most patients did not receive any antibiotic prophylaxis in the early years, prophylactic regimens consisting of oral penicillin or iv piperacillin/tazobactam or other combinations were administered later, however, inconsistently. In 2008, we commenced a prophylactic regimen with teicoplanin which was administered to almost all patients from the second half of 2009 on. The prophylactic regimen was started after myelosuppressive chemotherapy at the onset of severe neutropenia (absolute neutrophil count (ANC) ≤ 500/μl); teicoplanin was administered at a dose of 15–20 mg/kg iv on alternate days in our outpatient clinic or by oncologic nurses of our external nursing service at home until ANC increased to more than 500/μl. Dosing on alternate days was only applied as part of the prophylactic regimen. In case of admission to the hospital (due to fever or other reasons), vancomycin at a standard dose of 40 mg/kg/day iv was usually given instead of teicoplanin for cost reasons. Teicoplanin/vancomycin prophylaxis was combined with piperacillin/tazobactam or oral ciprofloxacin in few cases. Vancomycin or teicoplanin serum levels were only assessed in selected cases such as renal impairment.

All patients received continuous *Pneumocystis jirovecii* prophylaxis with trimethoprim-sulfamethoxazole three times per week as well as oral nonabsorbable amphotericin B as candida prophylaxis and paromomycin for enteral decontamination. Systemic fungal prophylaxis with itraconazole, voriconazole, or liposomal amphotericin was administered during neutropenia. During neutropenic phases, the use of antiseptic mouthwash was advised. Administration of granulocyte-colony-stimulating factor (G-CSF) was not recommended. Febrile neutropenia was defined as a body temperature of greater than 38.0 °C that persisted for an hour or a single temperature of ≥38.5 °C combined with neutropenia. A diagnosis of bacterial sepsis required positive blood culture and systemic clinical signs of infection. Bacterial blood cultures were routinely obtained from patients presenting with fever or other signs of infection. Samples obtained from all lumina of the central venous catheter were inoculated into three culture vials (BD BACTEC Plus Aerobic/F, Plus Anaerobic Plus, Mycosis IC/F, Becton, Dickinson and Company, Sparks, USA). Peripheral blood cultures were not routinely obtained. Toxicity grade of fever was defined based on the Common Terminology Criteria for Adverse Events v3.0, available online at http://ctep.cancer.gov/forms/. In case of fever, patients were admitted to the hospital and empiric broad-spectrum antibiotics (in most cases piperacillin/tazobactam in combination with aminoglycoside and glycopeptide) were started. Prophylactic or empiric administration of glycopeptides was mostly continued in case of negative gram-positive blood cultures. Routine surveillance conventional stool cultures were taken from all patients in the beginning of treatment and during follow-up.

### Statistical analysis

Fisher’s exact test and Wilcoxon test were used to compare courses without antibiotic prophylaxis and courses with teicoplanin/vancomycin prophylaxis. The statistical analysis was done with SAS System V9.2 (2008, SAS Institute, Cary, NC). All *p* values below 0.05 were considered significant.

## Results

Fifty patients were included in our study. Of these, 17 were treated according to the SR group and 31 according to HR arm. Of the remaining two, one patient with AML M3 received only an induction course with ADxE and continued therapy with ATRA and arsen trioxide only, while the other patient was diagnosed with secondary AML following treatment for acute lymphoblastic leukemia and received one course containing liposomal daunorubicin and fludarabine, followed by one course AI-2CDA, which was evaluated for our study. In total, 50 patients underwent 209 chemotherapy cycles, 10 of which (5 %) were excluded from our analysis (due to administration abroad *n* = 1, antibiotic treatment already along with chemotherapy *n* = 9). Of 199 evaluable courses, 42 contained low-dose cytarabine (ADxE *n* = 22, AIE *n* = 20), 89 intermediate-dose cytarabine (haM *n* = 43, AI *n* = 27, AI/2-CDA *n* = 19), and 68 high-dose cytarabine (HAM *n* = 29, HAE *n* = 34, HA *n* = 5, Table 2).

Ninety-eight of evaluable chemotherapy courses (49 %) were given with antibiotic prophylaxis using teicoplanin/vancomycin (± other: piperacillin/tazobactam *n* = 6, oral ciprofloxacin *n* = 7). Notably, 85/98 courses were primarily teicoplanin-based, whereas during 13 courses, only vancomycin and no teicoplanin was given. No systemic antibiotic prophylaxis was administered following 79 evaluable chemotherapy courses (40 %). Twenty-two additional courses were recorded with various prophylactic regimens: seven courses were given with oral penicillin, ten courses with daily piperacillin/tazobactam iv, and in the remaining five courses, other antibiotic combinations were administered. As the latter three groups were rather small and inhomogeneous, they were excluded from statistical analysis and only the large two groups (no prophylaxis and teicoplanin/vancomycin prophylaxis) were statistically compared.

Results of infectious episodes according to prophylaxis regimen are shown in Table 1: of 98 cycles with prophylaxis with teicoplanin/vancomycin, no patient developed VGS sepsis in comparison to 12 cases of VGS sepsis among 79 cycles without antibiotic prophylaxis (0 vs. 15 %, *p* < 0.0001). In addition, also episodes of total febrile neutropenia were significantly less frequent in the teicoplanin/vancomycin group (44 % vs. no prophylaxis 82 %, *p* < 0.0001). In 53 of 79 cycles (67 %) without primary antibiotic prophylaxis, vancomycin/teicoplanin was administered with occurrence of febrile neutropenia or other clinical signs of infection. Altogether, we recorded 25 episodes (in 20 patients) of culture-proven bacterial sepsis during the 199 courses (13 %). Gram-positive bacteria were isolated in 60 % (total *n* = 15, VGS *n* = 14, *Staphylococcus epidermidis n* = 1). One patient even had two episodes of VGS sepsis during intensive chemotherapy. Gram-negative septicemias occurred in 40 % (total *n* = 10, *E. coli n* = 4, *Pseudomonas aeruginosa n* = 4, *Klebsiella pneumoniae n* = 1, *Fusobacteria n* = 1). Of these 25 bacterial sepsis episodes, 13 occurred during courses without antibiotic prophylaxis, ten after administration of teicoplanin/vancomycin prophylaxis (all gram-negative bacteria), and two episodes of VGS sepsis occurred despite prophylaxis with oral penicillin. Hence, the mere sepsis rate was not significantly different between courses with teicoplanin/vancomycin prophylaxis and without antibiotic prophylaxis (*p* = 0.26, see Table 1). Sepsis started on average on day 15 from beginning of chemotherapy cycle after teicoplanin/vancomycin prophylaxis (median day 15, range 9–19) and on day 14 when no prophylaxis was administered (median 14, range 11–18; *p* = 0.32).

**Table 1:** Incidence and severity of infection according to prophylactic regimen

	Number	Viridans sepsis (%)	*p* value	Bacterial sepsis (%)	*p* value	Febrile neutropenia (%)	*p* value	Absolute days of fever >38C^a^ (median/mean)	*p* value	Toxicity grade of fever^a^ (median/mean)	ICU (*n*)	*p*
No prophylaxis	79	12 (15)	<0.0001	13 (16)	NS	65 (82)	<0.0001	4/6.1	<0.0001	2/2	4	0.038
Teico/vanco±	98	0		10 (10)		43 (44)		2/ 3.7		2/2	0	
Penicillin po	7	2 (29)		2 (29)		4 (57)		4/4.5		2/2	1	
Piperacillin/tazobactam	10	0		0		4(40)		4/3.8		1.5/ 1.5	0	
Other	5	0		0		0		NA		NA	0	
Total	199	14		25		116		4/5.1		2/2	5	

*NS* not significant

^a^In case of febrile neutropenia

Life-threatening complications occurred in five cases of bacterial sepsis (20 %), causing admission to intensive care unit (ICU) for treatment—in two of these episodes, VGS were isolated. In total, 14 % of patients with VGS sepsis (2/14) had to be treated at ICU. No patient in the study period died from infectious complications. Interestingly, severity of infection seemed to be worse in case of febrile neutropenia when no antibiotic prophylaxis had been administered with significantly more days of fever and higher rate of treatment at ICU (teicoplanin/vancomycin: median 2 days of fever, mean 3.7 vs. no prophylaxis: 4 and 6.1 days, *p* < 0.0001; ICU 0/98 [0 %] vs. 4/79 [5 %], *p* = 0.038).

We also compared sepsis rate and febrile neutropenia within the different cytarabine dose level subgroups. After intermediate-dose cytarabine courses, VGS sepsis occurred in 10 % (9/89) and in 7.4 % after high-dose courses (5/68, *p* = not significant), whereas no VGS case was found after the first induction (Table 2). The effect of teicoplanin/vancomycin prophylaxis appeared most impressing in the intermediate-dose group, resulting in a significantly lower rate of VGS sepsis (0 vs. 24 %, *p* = 0.0004) and febrile neutropenia (39 vs. 86 %, *p* < 0.0001) compared to no prophylaxis. However, a trend toward fewer VGS episodes (0 vs. 13 %, *p* = NS) and definitely also regarding less frequent febrile neutropenia (43 vs. 79 %, *p* = 0.008) was also seen in the high-dose cytarabine group. Only following the first induction course that the rate of febrile neutropenia was similar, whether teicoplanin/vancomycin prophylaxis was given or not (60 vs. 78 %, *p* = 0.45).

**Table 2:** Incidence and severity of infection according to different prophylactic regimen in different cytarabine dose level subgroups

Cytarabine dose level	Prophylaxis	Number	Viridans sepsis (%)	*p* value	Bacterial sepsis (%)	*p* value	Febrile neutropenia (%)	*p* value	Absolute days of fever >38C^a^ (median/mean)	*p* value	Toxicity grade of fever^a^ (median/mean)	ICU (*n*)	*p* value
Low dose (ADxE, AIE)	No	18	0	NA	0	NA	14 (78)	NS	8/8.9 (1–19)	NS	2/2.2	2	NS
	Teico/vanco ±	15	0		0		9 (60)		3/5.7 (1–15)		2/2	0	
	Other	9	0		0		3 (33)		5/4.3		1/1.3	0	
	Total	42	0		0		26 (62)		5.5/7.2		2/1.9	2	
Intermediate dose (haM, AI, AI-2CDA)	No	37	9 (24)	0.0004	10 (27)	NS	32 (86)	<0.0001	4/5.5 (1–21)	<0.0001	2/1.9	1	NS
	Teico/vanco±	46	0		6 (13)		18 (39)		2/2.9 (1–9)		2/2	0	
	Other	6	0		0		2 (33)		5.5/5.5		2.5/2.5	0	
	Total	89	9 (10)		16 (18)		52 (58)		3.5/4.6 (1–21)		2/2	0	
High dose (HAM, HAE, HA)	No	24	3 (13)	NS	3 (13)	NS	19 (79)	0.008	4/5	0.003	2/2.1	1	NS
	Teico/vanco±	37	0		4 (11)		16 (43)		3/3.3		2/2.2	0	
	Other	7	2 (29)		2 (29)		3 (43)		2/3		1/1.7	1	
	Total	68	5 (7)		9 (13)		38 (56)		3.5/4.1		2/2.1	2	

*NS* not significant, *NA* not applicable

^a^In case of febrile neutropenia

Data from hygiene surveillance were available until the end of 2015 for vancomycin-resistant enterocci (VRE) and the end of 2012 for VGS: routine surveillance stool cultures showed a low incidence of VRE isolates in our hospital (32 of 2071 isolates from 2006 to 2015, 1.5 %). In total, 797 enterococcus strains were isolated within the oncology department, 14 were vancomycin resistant (1.8 %). Only one of these 14 cultures was isolated from a patient with (secondary) AML following treatment for acute lymphoblastic leukemia (ALL). In this patient, surveillance cultures were already positive for the same VRE isolate with beginning of ALL therapy and can hence not be associated with our antibiotic prophylaxis. Altogether, we did not appreciate a rise in VRE incidence in the oncology department since the commencement of our prophylactic regimen with teicoplanin/vancomycin since 2008 (five VRE isolates between 2006 and 2009, five VRE isolates between 2009 and 2012, four VRE isolates between 2013 and 2015). Among all VGS isolates in the hospital (*n* = 750 between 2006 and 2012), not a single strain with teicoplanin or vancomycin resistance was detected. Of 88 strains which were tested for oral penicillin resistance, 19 were resistant (22 %).

## Discussion

Intensive chemotherapy followed by profound and prolonged neutropenia harbors a substantial risk for infectious complications among pediatric patients with AML [1, 8]. In particular, infection with VGS has been reported as a major cause for sepsis and pneumonia associated with considerable morbidity with high rates of ICU admission and even mortality in this cohort [4, 8, 22]. In order to prevent neutropenic sepsis, and VGS sepsis in particular, different approaches in terms of antibiotic prophylaxis have been considered [11, 23]. The Oregon Health and Science University retrospectively studied the use of iv vancomycin and ceftazidime and later iv cefepime mono and could demonstrate low rates of infection and VGS sepsis, in particular [24]. At St. Jude Children’s Research center, a prophylactic regimen combining iv vancomycin with oral ciprofloxacin or a cephalosporin has been administered to pediatric AML patients in neutropenia. This regimen resulted in reduction of incidence of VGS septicemia and hospitalization days, however not of febrile neutropenia episodes [12, 13]. One disadvantage of vancomycin is a short half-life with the need of administration at least every 12 h over 1 to 2 h. In comparison, teicoplanin is a glycopeptide antibiotic with similar antibacterial spectrum and longer half-life that is usually administered once a day (6 mg/kg/day following a loading dose) iv shot. However, several previous studies used teicoplanin in a higher single dose (15–20 mg/kg/day) on alternate days/three times a week with similar clinical effectiveness and serum concentrations [14, 15]. In this retrospective analysis, we studied the effect of a prophylactic regimen with iv teicoplanin on alternate days (daily iv vancomycin in case of hospital admission) compared to no prophylactic antibiotics in a group of 50 pediatric AML patients who were treated on the AML-BFM 2004 protocol between 2005 and 2015. Prophylaxis with teicoplanin/vancomycin resulted in dramatic and significant decrease of VGS sepsis compared to no antibiotic prophylaxis (0/98, 0 %, vs. 12/79, 15 %, *p* < 0.0001) and significant reduction of febrile neutropenia episodes (43/98, 44 %, vs. 65/79, 82 %, *p* < 0.0001) and absolute fever days (*p* < 0.0001). This effect was also visible in the intermediate-dose cytarabine subgroups with significantly lower rate of VGS sepsis and febrile neutropenia following teicoplanin prophylaxis (0 vs. 24 %, *p* = 0.0004; 39 vs. 86 %, *p* < 0.0001; Table 2) and in the high-dose cytarabine subgroup for reduction of febrile neutropenia (43 vs. 79 %, *p* = 0.008). Of course, results in these subgroups have to be regarded with caution due to small numbers. Hence, our study supports the effectiveness and feasibility of preventive antibiotics in neutropenic pediatric AML patients.

High-dose cytarabine has been frequently claimed a risk factor for VGS sepsis in AML patients [25]. Retrospective data from study AML-BFM 93 indeed demonstrated a significantly higher VGS sepsis rate following high-dose cytarabine cycles whereas in a recent NOPHO-AML study and in the AML-BFM 2004 study, no significant difference of VGS sepsis rate was observed between lower-dose and high-dose courses [4, 9, 22]. In our study, rate of VGS sepsis was comparably high following intermediate-dose and high-dose cycles (9.3 and 7.4 %) while no VGS sepsis occurred after induction cycles with low-dose cytarabine.

VGS sepsis is known be associated with considerable morbidity and in case of viridans streptococcal shock syndrome high mortality [10]. VGS sepsis was found to be significantly more life-threatening in comparison to other infections according to data from the Children’s Cancer Group [25]. Recent data from international study groups also describe a high incidence of life-threatening complications, i.e., ICU admission, associated with VGS sepsis: while AML-BFM 2004 reported that 9.8 % of patients with VGS sepsis needed intensive care treatment, incidence of ICU treatment of VGS cases was 11.6 % within the NOPHO group [9, 22]. Life-threatening shock was observed in 13 % of neutropenic pediatric patients with VGS sepsis. In our retrospective study, 14 % of cases with VGS sepsis needed ICU treatment. With teicoplanin/vancomycin prophylaxis, we could see a significant reduction of incidence of ICU treatment in comparison to no prophylaxis (0/98, 0 %, vs. 4/79, 5 %, *p* = 0.038).

VGS prophylaxis with oral penicillin G for AML patients has been frequently discussed and was administered during seven chemotherapy cycles of our study period as well [23]. Although the numbers are small and have to be regarded with caution, rate of febrile neutropenia with penicillin prophylaxis was rather high (4/7, 57 %). More importantly, two patients developed VGS sepsis despite oral penicillin prophylaxis, of which one patient had to be treated at ICU for almost 2 weeks as she developed pneumonitis and cardiac insufficiency. In both cases, the antibiogramm demonstrated resistance to penicillin. According to hygienic surveillance data at St. Anna Children’s Hospital, 22 % of isolated VGS showed in vitro resistance to oral penicillin; however, rates of up to 40 % have been reported in other studies [22, 26]. Hence, VGS prophylaxis with oral penicillin cannot be generally recommended.

Among 98 patients of our study who received teicoplanin/vancomycin prophylaxis, 10 % developed bacterial sepsis, all with gram-negative bacteria. In contrast, bacterial sepsis among the patients without antibiotic prophylaxis was mainly associated with gram-positive bacteria/VGS. Hence, we considered if gram-negative sepsis might have been prevented in the group without antibiotic prophylaxis by early administration of broad-spectrum antibiotics with emerging VGS sepsis. However, our data show that sepsis occurred on similar days within the two groups (average/median: no prophylaxis: day 14/14, teicoplanin/vancomycin: day 15/15) which argues against this assumption. Hence, due to considerable morbidity of gram-negative sepsis, combination of teicoplanin/vancomycin with an antibiotic covering gram-negative spectrum should be considered.

Frequent handling of central venous catheters, even by trained caregivers in a home infusion setting, bears the risk of catheter-associated bloodstream infections. When patients were not admitted to our hospital, teicoplanin was administered on alternate days/three times per week iv shot either in our outpatient clinic or by nurses of our external oncologic nursing service at the patient’s home, resulting in minimal necessity to handle central catheters. Another major advantage of administration of antibiotics on an outpatient basis or even at home is a decreased need to stay in the hospital with increase in patient’s quality of life along with expected lower health care costs.

A major concern with application of antibiotic prophylaxis is the emergence of resistant bacteria strains. Patients with hematological malignancies are more prone to colonization and infection with VRE due to the routine use of broad-spectrum antibiotics, and antibiotic options in case of infection with VRE are limited [27]. Data from St. Jude Hospital demonstrated a higher incidence of VRE isolates in rectal cultures among the group that received prophylactic antibiotic therapy with intravenous vancomycin (± cefepime, ciprofloxacin, oral cephalosporin) with five episodes of VRE bacteremia [13]. Since the introduction of our prophylactic regimen with teicoplanin/vancomycin, we did not appreciate an increase in VRE isolates in our oncology department at St. Anna Children’s Hospital. Our data even show that many patients without primary glycopeptide prophylaxis eventually received glycopeptides as preemptive therapy in case of septicemia (67 % of all nonprophylaxis cycles). This might add to a potential selection pressure on VRE albeit not preventing morbidity from VGS. However, the incidence of resistant bacteria strains and possible higher incidence of fungal infections with antibiotic prophylaxis have to be closely monitored in the future. Benefits of antibiotic prophylaxis need to be continuously balanced against negative effects of such a regimen.

There are limitations to our study: we did not randomly assign patients to receiving teicoplanin/vancomycin prophylaxis or not but performed a retrospective chart review to document infections. Prospective controlled randomized studies, preferably involving multiple international institutions due to the low absolute number of pediatric AML patients, are needed in the future to confirm our results.

## Conclusions

Antibiotic prophylaxis with teicoplanin (or vancomycin) appears safe and feasible and resulted in dramatic reduction of VGS-associated sepsis and decreased incidence of febrile neutropenia in neutropenic pediatric patients with AML. The possibility to administer teicoplanin on alternate days on an outpatient basis or at home could contribute to patient’s quality of life and decrease health care costs. Multi-institutional studies are needed to evaluate this prophylactic regimen in a larger cohort and acquire more data on the development of possible bacterial resistance.

## Electronic supplementary material

Below is the link to the electronic supplementary material.

ESM 1 (PDF 201 kb)
