# Supplementary material for: Antibiotic prophylaxis with teicoplanin on alternate days reduces rate of viridans sepsis and febrile neutropenia in pediatric patients with acute myeloid leukemia
Source: Ann Hematol. 2016 Oct 4;96(1):99–106. doi: 10.1007/s00277-016-2833-5 (PMC5225941; doi:10.1007/s00277-016-2833-5)
Supplement: Supplementary file 1 — (PDF 201 kb) [file 277_2016_2833_MOESM1_ESM.pdf]

Annals of Hematology

Antibiotic prophylaxis with teicoplanin on alternate days reduces rate of viridans sepsis and febrile neutropenia in pediatric patients with acute myeloid leukemia

Heidrun Boztug, Nora Mühlegger, Ulrike Pötschger, Andishe Attarbaschi, Christina Peters, Georg Mann, Michael Dworzak
Correspondence: Heidrun Boztug and Michael Dworzak; St. Anna Kinderspital and Children´s Cancer Research Institute, Department of Pediatrics, Medical University of Vienna; E-mail: heidrun.boztug@stanna.at and michael.dworzak@stanna.at

Supplemental Table 1. Chemotherapy cycles in AML-BFM 2004

		Dose, mg/sqm/dose	Days	Total dose of cytarabine (mg/sqm)
**Induction 1**	**ADxE**			
	Cytarabine	100 as continuous infusion over 48 hours	1,2	1400
	Cytarabine	100 every 12 hours	3-8	
	Etoposide	150	6,7,8	
	**AIE**			
	Cytarabine	100 as continuous infusion over 48 hours	1,2	1400
	Cytarabine	100 every 12 hours	3-8	
	Etoposide	150	6,7,8	
	Idarubicine	12	3,5,7	
**Induction 2**	**HAM (HR only)**			
	Cytarabine	3000 every 12 hours	1-3	18000
	Mitoxantrone	10	3,4	
	**AI (SR only)**			
	Cytarabine	500 as continuous infusion	1-4	2000
	Idarubicine	7	3,5	
**Consolidation 1**	**AI/2-CDA (HR only)**			
	Cytarabine	500 as continuous infusion	1-4	2000
	Idarubicine	7	3,5	
	2-CDA	6	1,3	

**Consolidation 2**	**haM**			
	Cytarabine	1000 every 12 hours	1-3	6000
	Mitoxantrone	10	3,4	
**Intensification**	**HAE**			
	Cytarabine	3000 every 12 hours	1-3	18000
	Etoposide	125	2,3,4,5	
